# Association Between Third-Trimester Ultrasonography With Histopathological Changes in the Placenta Among Mothers With Gestational Diabetes Mellitus

**DOI:** 10.7759/cureus.91574

**Published:** 2025-09-03

**Authors:** Madasu Naga Venkata Gnana Anusha, Minthami Sharon P, Kavitha Washington

**Affiliations:** 1 Obstetrics and Gynaecology, Sree Balaji Medical College and Hospital, Chennai, IND

**Keywords:** fetal development, gestational diabetes mellitus (gdm), placenta, placental histopathology, the amniotic fluid index

## Abstract

Background

Gestational diabetes mellitus (GDM) is a prevalent metabolic disorder during pregnancy, associated with significant maternal and fetal complications. GDM affects placental structure and function, potentially compromising fetal development. Third-trimester ultrasonography (USG) serves as a non-invasive tool for fetal and placental assessment, while histopathological examination provides definitive evidence of placental changes. This study aimed to evaluate the correlation between third-trimester USG findings and placental histopathological changes in GDM pregnancies.

Methodology

A hospital-based observational study was conducted over 18 months at a tertiary care center in Chennai. A total of 85 pregnant women diagnosed with GDM after 28 weeks of gestation were included. All participants underwent third-trimester USG to assess placental thickness, estimated fetal weight (EFW), amniotic fluid index (AFI), and placental grading. After delivery, placentas were collected and examined histopathologically for changes such as syncytial knots, chorangiosis, fibrinoid necrosis, and intervillous thrombosis. Data were analyzed using SPSS (IBM SPSS Statistics for Windows, IBM Corp., Version 26, Armonk, NY), and associations between USG and histopathological findings were assessed using independent t-tests.

Results

A total of 85 women with gestational diabetes were studied. Nearly half were aged 26-30 years (42, 49.4%), while 20 (23.5%) were 31-35 years. Management was most commonly with oral hypoglycemic agents (40, 47.1%), followed by medical nutrition therapy (21, 24.7%). Antenatal steroids were administered in 38 (44.8%). Most USG examinations were at 31-32 weeks (40, 47.1%). Term deliveries (37-38 weeks) occurred in 77 (90.6%), with 52 (61.2%) vaginal and 33 (38.8%) caesarean births. Placental grading showed Grade 3 in 78 (91.7%). Histopathology frequently revealed syncytial knots, chorangiosis, fibrinoid necrosis, and intervillous thrombosis, significantly associated with increased placental thickness and EFW.

Conclusion

Third-trimester USG findings, particularly increased placental thickness and EFW, correlate significantly with histopathological changes in GDM placentas. These findings underscore the value of antenatal USG as a predictive tool for placental pathology and potential adverse outcomes in GDM pregnancies.

## Introduction

Gestational diabetes mellitus (GDM) is defined as glucose intolerance with onset or first recognition during the second or third trimester of pregnancy, excluding pre-existing diabetes [1]. This timing reflects the increasing influence of placental hormones that induce insulin resistance in later gestation. GDM is the most common metabolic disorder in pregnancy and contributes significantly to maternal and neonatal morbidity. Reported prevalence varies widely, from 6.8% to 40.3%, influenced by geographic, ethnic, and socioeconomic factors, as well as differing diagnostic criteria [2,3]. Such variability highlights the need for population-specific screening protocols and individualized clinical management. The hyperglycemic intrauterine environment increases the risk of complications, including macrosomia, preeclampsia, and neonatal hypoglycemia, while also predisposing both mother and child to long-term metabolic disorders if glycemic control is suboptimal.

Poorly controlled GDM is associated with a spectrum of adverse pregnancy outcomes. Maternal complications include increased rates of hypertensive disorders, polyhydramnios, and operative deliveries, while fetal complications comprise macrosomia, preterm birth, neonatal hypoglycemia, hyperbilirubinemia, and respiratory distress syndrome (RDS) [4,5]. These complications not only impact immediate perinatal health but may also have lasting consequences. Women diagnosed with GDM face a substantially elevated lifetime risk of developing type 2 diabetes mellitus (T2DM) and cardiovascular diseases, and studies have shown that up to 50% may develop T2DM within 10 years of the index pregnancy [6]. Moreover, their offspring are predisposed to early-onset obesity, impaired glucose tolerance, and metabolic syndrome later in life, suggesting an intergenerational transmission of metabolic risk [7].

Central to pregnancy and fetal development, the placenta functions as a critical interface between the mother and the fetus. It regulates nutrient and gas exchange, hormone production, and waste elimination, and plays essential roles in immune modulation. Structurally, the placenta continuously adapts throughout gestation to support fetal demands [8]. In GDM pregnancies, however, this adaptive process is often disrupted due to maternal hyperglycemia. Numerous placental structural and functional abnormalities have been documented in GDM, including villous immaturity, increased syncytial knots, chorangiosis, fibrinoid necrosis, and abnormal vasculature. These changes are believed to stem from a complex interplay of oxidative stress, inflammation, endothelial dysfunction, and impaired angiogenesis triggered by persistent hyperglycemia. The exact pathophysiological mechanisms remain under investigation, but current evidence points toward glycemic dysregulation as a major contributor to placental pathology.

Third-trimester ultrasonography (USG) plays a pivotal role in the antenatal monitoring of pregnancies complicated by GDM. It provides crucial information on fetal growth and well-being and helps identify potential complications early. Parameters, such as estimated fetal weight (EFW), amniotic fluid index (AFI), placental grade, and cerebroplacental ratio (CPR), are routinely assessed. GDM is frequently associated with elevated EFW due to fetal hyperinsulinemia, leading to macrosomia, which increases the risk of shoulder dystocia and birth trauma. Likewise, AFI is often elevated due to fetal polyuria, a result of high glucose levels in the amniotic fluid [9]. Abnormalities in placental maturity, including delayed maturation or calcification, may reflect functional insufficiency. Altered CPR values may suggest fetal hypoxia, necessitating timely intervention. Therefore, USG not only aids in fetal surveillance but also informs decisions regarding timing and mode of delivery.

Histopathological evaluation of the placenta offers valuable postnatal insights into the intrauterine environment. In GDM, placental pathology may show increased placental weight and thickness, reflecting compensatory hypertrophy. Microscopically, features such as excessive vascular proliferation, increased syncytial knots, intervillous fibrin deposition, and infarcts are frequently noted [10]. However, these findings are not universal. A subset of placentas from GDM pregnancies appears histologically normal, suggesting that the degree of placental change may be influenced by the level of maternal glycemic control, duration of exposure, and presence of other maternal comorbidities such as hypertension or obesity [7]. This inter-individual variability highlights the importance of correlating antenatal imaging with histopathological outcomes for a comprehensive understanding.

In India, the burden of GDM is rising, particularly in urban regions, largely due to lifestyle factors such as increased adiposity, dietary transitions, and reduced physical activity. In South India, including Tamil Nadu, prevalence rates ranging from 17% to 21% have been reported, making it a major public health concern [11]. The increasing trend has led to the widespread adoption of universal screening strategies using oral glucose tolerance tests (OGTT) and integrated antenatal follow-up, including third-trimester USG evaluations [12]. Given this background, the present study was designed to systematically evaluate and correlate third-trimester ultrasonographic findings with histopathological changes in the placenta among pregnant women diagnosed with GDM.

## Materials and methods

Study design and setting

This cross-sectional analytical study was conducted over a period of one and a half years, from January 2023 to June 2024, in the Department of Obstetrics and Gynaecology at a tertiary care medical college and teaching hospital in Chennai, Tamil Nadu, India. The study aimed to evaluate the correlation between third-trimester ultrasonographic findings and histopathological changes in the placenta among women diagnosed with GDM. It was carried out in collaboration with the Department of Pathology, which undertook the histological examination of placental tissue. The study commenced after obtaining formal approval from the Institutional Human Ethics Committee of Sree Balaji Medical College and Hospital (SBMCH), Chennai (reference number: 002/SBMCH/IHEC/2023/2043). Ethical principles outlined in the Declaration of Helsinki were followed, and all participants were enrolled after providing written informed consent.

Study population and sampling

The study included pregnant women attending the outpatient and inpatient services of the Department of Obstetrics and Gynaecology who were diagnosed with GDM after 28 weeks of gestation, consistent with the typical timing of GDM detection in clinical practice. Participants were selected using purposive sampling based on predefined criteria. Inclusion criteria were women with confirmed GDM beyond 28 weeks, those who received antenatal care and delivered at the study hospital, and those who provided informed consent. Exclusion criteria were gestational age < 28 weeks, non-diabetic pregnancies, and comorbidities such as preeclampsia, intrauterine growth restriction (IUGR), and major fetal anomalies. These criteria ensured uniformity in the study population and reduced confounding, though they may limit the generalizability of findings to broader obstetric populations.

Sample size calculation

The sample size was calculated using a correlation coefficient (r) of 0.3, which reflects a moderate relationship between ultrasonographic parameters and histopathological changes in the placenta from a previous study [13]. Using the statistical formula for correlation coefficient, where Zα = 1.96 for a 95% confidence interval, Zβ = 0.841 for 80% power, and C = 0.3095 (derived from Fisher’s Z transformation of r = 0.3), the final estimated sample size was 85 participants.

Diagnosis of GDM and recruitment

GDM was diagnosed using the one-step Oral Glucose Challenge Test (OGCT) following the Diabetes in Pregnancy Study Group India (DIPSI) guidelines [14]. According to DIPSI, a plasma glucose value ≥140 mg/dL, measured two hours after ingestion of 75 g of oral glucose (regardless of fasting status), is considered diagnostic of GDM. Eligible women were identified during their antenatal visits or hospital admissions. Those who fulfilled the inclusion criteria and consented were enrolled consecutively into the study. Recruitment was carried out after a detailed explanation of the study objectives and procedures, and confidentiality of patient data was assured.

Data collection and clinical assessment

Following recruitment, participants underwent a comprehensive interview using a pre-tested, semi-structured proforma. This form was used to collect sociodemographic information, including age, education level, and socioeconomic status (classified using the Modified BG Prasad Scale). Detailed obstetric history, including gravidity, parity, previous obstetric outcomes (such as history of GDM, macrosomia, or stillbirth), and current pregnancy course, was documented. Clinical records were reviewed to extract data on antenatal complications, number of antenatal visits, medication use, and hospitalizations. Additional details regarding GDM management (dietary control, use of oral hypoglycemic agents (OHA), or insulin therapy), administration of antenatal corticosteroids, and monitoring strategies were also collected. The gestational age at delivery, mode of delivery (vaginal or caesarean), neonatal birth weight, and immediate postnatal outcomes were noted.

Ultrasonographic assessment

All participants underwent a detailed third-trimester ultrasonographic scan between 32 and 37 weeks of gestation as part of routine antenatal care. The ultrasound examinations were performed by qualified radiologists using high-resolution ultrasound equipment (GE Voluson (GE HealthCare, Chicago, IL) or equivalent). The radiologists were blinded to the patients' clinical and biochemical information to minimize observer bias. The ultrasonographic parameters recorded included EFW, AFI, placental thickness and grade, and the CPR. EFW was calculated using Hadlock’s formula, which incorporates measurements such as biparietal diameter, abdominal circumference, and femur length. The AFI was determined using the four-quadrant method; a value above 24 cm was considered indicative of polyhydramnios. Placental grading was performed according to Grannum’s classification system, which assesses placental maturity based on calcification patterns. The CPR was computed as the ratio of the pulsatility index (PI) of the middle cerebral artery (MCA) to that of the umbilical artery (UA), with a value less than one considered abnormal and suggestive of fetal compromise.

Placental collection and histopathology

All participants were followed up until delivery. Immediately after delivery, the placenta was collected under aseptic precautions, rinsed in normal saline, and preserved in 10% buffered formalin. Each sample was labelled with the participant’s unique identification number and delivery details. The fixed placental specimens were then transported to the Department of Pathology for gross and microscopic examination. On gross inspection, placental weight, thickness, and any macroscopic abnormalities such as infarcts or calcifications were recorded. For histological analysis, representative tissue sections from the central and peripheral regions of the placenta, as well as from the umbilical cord and membranes, were embedded in paraffin blocks and stained with hematoxylin and eosin (H&E). The stained slides were examined under a light microscope by an experienced pathologist who was blinded to the ultrasound and clinical data. Microscopic findings assessed included villous immaturity or hypermaturity, presence of excessive syncytial knots, chorangiosis (increased capillary proliferation in terminal villi), intervillous fibrin deposition, infarction, fibrinoid necrosis, thrombosis, and features of maternal vascular malperfusion such as decidual vasculopathy. In our histopathological assessment, the term vasculopathy was used to denote an overarching vascular injury pattern within the placenta. For this study, vasculopathy specifically included features such as fibrinoid necrosis of spiral arteries, atherosis, and evidence of maldevelopment or narrowing of villous vasculature.

Data management and statistical analysis

The collected data were entered and organized using Microsoft Excel 2019 (Microsoft® Corp., Redmond, WA) and subsequently analyzed using SPSS (IBM SPSS Statistics for Windows, IBM Corp., Version 26, Armonk, NY). Descriptive statistics were used to summarize the demographic and clinical characteristics of the study participants. Continuous variables, such as maternal age, AFI, EFW, and placental thickness, were expressed as means with standard deviations, while categorical variables such as mode of delivery, placental grade, and histopathological findings were summarized using frequencies and percentages. The independent sample t-test was employed to compare mean values between groups with and without specific histological changes. A p-value of less than 0.05 was considered statistically significant in all tests.

## Results

The present study was conducted among 85 pregnant women with gestational diabetes attending the outpatients and inpatient Department of Obstetrics and Gynaecology in a tertiary medical college in Chennai. The majority of the study participants were aged between 26 and 30 years, comprising 42 (49.4%) women, followed by 20 (23.5%) in the 31-35 age group, 17 (20.0%) aged 20-25 years, and six (7.1%) aged 36-40 years. Regarding treatment for GDM, 40 (47.1%) participants were managed with OHA, 21 (24.7%) received medical nutrition therapy (MNT), 14 (16.4%) were treated with a combination of OHA and insulin, and 10 (11.8%) received insulin alone. Antenatal corticosteroids were administered in 38 (44.8%) women, while 47 (55.2%) did not receive steroids. At the time of ultrasound in the third trimester, 40 (47.1%) women were between 31 and 32 weeks of gestation, 33 (38.8%) were between 28 and 30 weeks, and 12 (14.1%) were between 33 and 34 weeks. The majority were delivered vaginally, with 52 (61.2%) undergoing normal vaginal delivery, while 33 (38.8%) had a lower segment caesarean section (LSCS). Most deliveries occurred at 37-38 weeks of gestation in 77 (90.6%) women, while eight (9.4%) delivered between 35 and 36 weeks. On ultrasonographic evaluation, placental grading showed Grade 3 maturity in 78 (91.7%) cases and Grade 2 in seven (8.3%) cases (Table 1).

**Table 1:** Distribution of Study Participants Based on Sociodemographic, Treatment, and Pregnancy Details (n = 85)

Variable	Category	Frequency n (%)
Age (in Years)	20-25	17 (20.0%)
	26-30	42 (49.4%)
	31-35	20 (23.5%)
	36-40	6 (7.1%)
Treatment for GDM	Medical nutrition therapy (MNT)	21 (24.7%)
	Oral hypoglycemic agents (OHA)	40 (47.1%)
	OHA + insulin	14 (16.4%)
	Insulin alone	10 (11.8%)
Antenatal Steroid Use	Administered	38 (44.8%)
	Not administered	47 (55.2%)
Gestational Age at USG	28-30 weeks	33 (38.8%)
	31-32 weeks	40 (47.1%)
	33-34 weeks	12 (14.1%)
Mode of Delivery	Normal vaginal delivery	52 (61.2%)
	Lower segment cesarean section (LSCS)	33 (38.8%)
Gestational Age at Delivery	35-36 weeks	8 (9.4%)
	37-38 weeks	77 (90.6%)
Placental Grading on USG	Grade 2	7 (8.3%)
	Grade 3	78 (91.7%)

The mean gestational age at delivery of the study participants was 37.21 + 0.785 years; 90.4% of the mothers delivered at term (37-38 weeks), while 9.4% were preterm deliveries. Mean placental weight was 616.26 ± 62.85 g, with the highest being 780 g and the lowest being 540 g.

**Figure 1:**
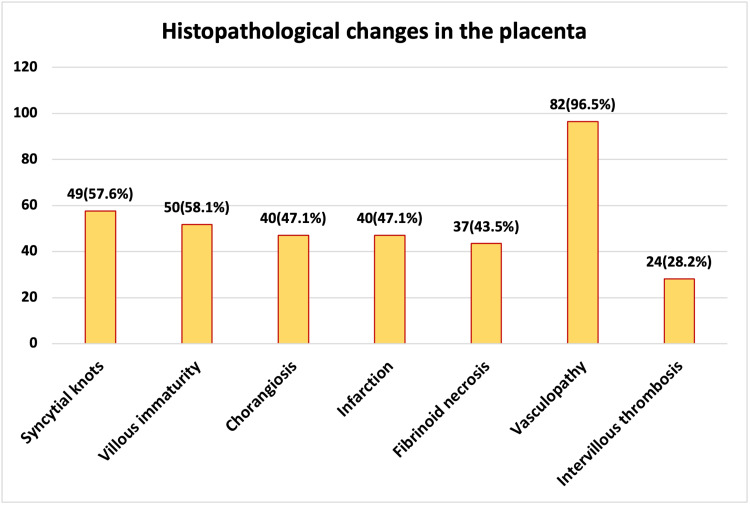
Distribution of Study Subjects Based on Histopathological Changes in the Placenta (n = 85)

Based on the histopathological evaluation of placental samples from 85 participants, vasculopathy was the most commonly observed change, found in 82 (96.5%) cases. Villous immaturity was present in 50 (58.1%) placentas, closely followed by syncytial knots in 49 (57.6%) cases. Both chorangiosis and infarction were identified in 40 (47.1%) cases each. Fibrinoid necrosis was noted in 37 (43.5%) of the samples, while intervillous thrombosis was observed in 24 (28.2%) cases. These findings suggest a high prevalence of vascular and developmental abnormalities in the placentas of women diagnosed with GDM (Figure 1).

Table 2 shows significant associations between specific placental histopathological changes and third-trimester ultrasonographic findings. In cases with syncytial knots, the mean placental thickness was significantly higher at 36.34 ± 4.66 mm compared to 33.96 ± 3.66 mm in those without syncytial knots (p = 0.013). The EFW was also significantly higher in these cases (3180.45 ± 450.94 g) than in those without (2887.31 ± 350.58 g, p = 0.002). However, there was no significant difference in AFI (16.76 ± 6.59 cm vs. 15.00 ± 4.83 cm, p = 0.179). For chorangiosis, placental thickness was significantly greater at 37.01 ± 5.03 mm compared to 33.84 ± 3.13 mm in those without the lesion (p = 0.001). EFW was also elevated (3220.83 ± 437.47 g vs. 2910.04 ± 379.50 g, p = 0.001), and AFI was higher in affected cases (17.94 ± 6.65 cm) versus unaffected cases (14.30 ± 4.67 cm, p = 0.004). In patients with fibrinoid necrosis, placental thickness was significantly higher (37.05 ± 4.61 mm) compared to those without (34.01 ± 3.78 mm, p = 0.001), and EFW was higher (3186.81 ± 404.88 g) than in those without necrosis (2955.69 ± 433.41 g, p = 0.014). AFI was slightly higher (16.59 ± 6.46 cm) in affected placentas but not statistically significant (p = 0.433). Finally, intervillous thrombosis showed strong associations with all three USG parameters. Placental thickness was markedly increased (38.91 ± 4.76 mm) compared to 33.93 ± 3.38 mm in those without thrombosis (p < 0.001). EFW was significantly higher (3337.13 ± 474.99 g) than in unaffected cases (2945.80 ± 365.59 g, p < 0.001), and AFI was also greater (18.10 ± 6.43 cm vs. 15.19 ± 5.58 cm, p = 0.042).

**Table 2:** Association of Histopathological Changes in the Placenta With the Third-Trimester USG Finding *Independent samples t-test. p-value < 0.05 - statistically significant. Placental thickness (mm). AFI - amniotic fluid index (cm); EFW - estimated fetal weight (g)

Variables			Mean	SD	t-value	p-value
Syncytial Knots	Placental thickness	Present	36.341	4.666	2.538	0.013*
		Absent	33.961	3.66		
	EFW	Present	3180.45	450.944	3.244	0.002*
		Absent	2887.31	350.582		
	AFI	Present	16.757	6.587	1.355	0.179
		Absent	15	4.825		
Chorangiosis	Placental thickness	Present	37.01	5.032	3.526	0.001*
		Absent	33.842	3.13		
	EFW	Present	3220.83	437.472	3.507	0.001*
		Absent	2910.04	379.496		
	AFI	Present	17.935	6.649	2.939	0.004*
		Absent	14.304	4.667		
Fibrinoid Necrosis	Placental thickness	Present	37.054	4.606	3.348	0.001*
		Absent	34.006	3.784		
	EFW	Present	3186.81	404.878	2.508	0.014*
		Absent	2955.69	433.406		
	AFI	Present	16.592	6.46	0.787	0.433
		Absent	15.567	5.53		
Intervillous Thrombosis	Placental thickness	Present	38.908	4.756	5.429	<0.001*
		Absent	33.926	3.375		
	EFW	Present	3337.13	474.995	4.071	<0.001*
		Absent	2945.8	365.586		
	AFI	Present	18.096	6.426	2.068	0.042*
		Absent	15.193	5.578		

## Discussion

This study aimed to assess the correlation between third-trimester USG parameters and histopathological changes in the placenta among women diagnosed with GDM. The findings from this cross-sectional study provide compelling evidence that structural and vascular alterations in the placenta - often linked to hyperglycemic insults - can be anticipated using non-invasive imaging tools like USG, particularly by assessing placental thickness, EFW, and AFI.

The most striking observation was the high prevalence of vasculopathy (96.5%), along with syncytial knots (57.6%), villous immaturity (58.1%), chorangiosis (47.1%), and fibrinoid necrosis (43.5%). These findings reflect significant maternal-placental vascular pathology, possibly due to prolonged exposure to hyperglycemia, leading to oxidative stress, endothelial dysfunction, and impaired angiogenesis. Previous studies have established that GDM is associated with placental hypoxia and compensatory angiogenesis, which manifest histologically as chorangiosis and increased syncytial knots. Mayhew et al. [15] demonstrated that even modest hyperglycemia could disrupt normal villous development, resulting in villous immaturity and excess syncytial knot formation, consistent with the findings of our study.

Ultrasound parameters, such as increased EFW, AFI, and placental thickness, were positively correlated with histopathological abnormalities, suggesting that these imaging features may act as early markers of placental stress. Notably, fibrinoid necrosis and intervillous thrombosis, which were observed in 43.5% and 28.2% of placentas, respectively, were significantly associated with increased placental thickness and fetal overgrowth. These abnormalities suggest impaired maternal-fetal perfusion and chronic hypoxic insult. Daskalakis et al. [16] had earlier emphasized that intervillous thrombi are not only markers of poor placental perfusion but also correlate with adverse outcomes like preterm delivery and low Apgar scores in diabetic pregnancies. Hence, their presence should prompt clinicians to anticipate potential perinatal risks.

Our study further underscores the importance of placental thickness as a potential indicator of histological abnormalities. In our cohort, most patients had a Grade 3 placenta on USG, and increased thickness was often associated with villous immaturity, vascular lesions, and chorangiosis. Sharma et al. [17] reported a similar association, suggesting that abnormal placental thickness on USG in GDM pregnancies should raise suspicion for underlying histopathological changes. These findings support the idea that USG, when used alongside clinical and metabolic data, can help identify high-risk pregnancies requiring closer surveillance.

From a pathophysiological standpoint, GDM-induced placental changes may be explained by persistent maternal hyperglycemia, which triggers a cascade of inflammatory and vascular responses. Hyperglycemia promotes oxidative stress and alters cytokine levels, leading to endothelial cell damage and capillary proliferation. This results in excessive deposition of fibrin, necrosis, thrombosis, and altered villous architecture, all of which were evident in the present study. While these histological lesions are compensatory in nature to maintain adequate fetal oxygenation and nutrient exchange, their persistence or severity may impair placental efficiency and fetal well-being.

Clinically, few findings suggest that third-trimester USG serves as a valuable tool in predicting underlying placental pathology in GDM pregnancies [18,19]. Identifying increased placental thickness, elevated AFI, or accelerated fetal growth should alert clinicians to the possibility of placental insufficiency or maladaptation. Moreover, antenatal decisions such as the timing and mode of delivery can be better informed when imaging findings are integrated with histological knowledge. Postnatally, examining the placenta, especially in cases of macrosomia, stillbirth, or unexpected complications, could yield insights into placental dysfunction and guide counselling for future pregnancies.

However, this study is not without limitations. First, the use of a purposive sampling technique may introduce selection bias and limit the generalizability of the findings. The study was hospital-based and might reflect a referral bias wherein more complicated cases of GDM were included. Second, we did not quantify glycemic control parameters such as fasting blood glucose, postprandial values, or HbA1c levels, which could have provided insight into the correlation between glycemic burden and placental pathology. Finally, the sample size, although statistically justified, could be considered modest for subgroup analyses based on treatment modality or severity of GDM.

The implications of this study are multifold. It emphasizes the clinical utility of third-trimester USG in GDM pregnancies as a non-invasive, real-time tool to anticipate placental maladaptation. For obstetricians, this can facilitate timely intervention and improve perinatal outcomes. Moreover, histopathological examination of the placenta, although often overlooked in routine practice, can offer critical insights into in-utero events, guide management in future pregnancies, and support legal and ethical documentation in adverse outcomes. Future research should aim to establish a placental risk score combining ultrasonographic and histological variables, possibly integrated with glycemic control metrics, to comprehensively stratify GDM pregnancies.

## Conclusions

This study highlights a significant association between third-trimester ultrasonographic findings and histopathological changes in the placenta among women with GDM. Increased placental thickness, EFW, and AFI on ultrasound were strongly correlated with specific placental lesions such as syncytial knots, villous immaturity, chorangiosis, fibrinoid necrosis, and intervillous thrombosis. These lesions likely represent downstream effects of chronic hyperglycemia and placental vascular stress, whereas vasculopathy, defined by maternal vascular malperfusion changes such as decidual arteriopathy, fibrinoid necrosis of spiral arteries, and atherosis, should be considered a distinct primary vascular abnormality. The findings underscore the utility of third-trimester ultrasound as a non-invasive predictor of placental dysfunction in GDM, while post-delivery histopathology provides confirmatory evidence and aids in counselling for future pregnancies. Integration of antenatal imaging with pathological evaluation thus offers a more comprehensive framework for the management of GDM, with the potential to improve maternal and neonatal outcomes.
